# Network analysis and juvenile idiopathic arthritis (JIA): a new horizon for the understanding of disease pathogenesis and therapeutic target identification

**DOI:** 10.1186/s12969-016-0078-4

**Published:** 2016-07-02

**Authors:** Rachelle Donn, Chiara De Leonibus, Stefan Meyer, Adam Stevens

**Affiliations:** Musculoskeletal Research Group, The Centre For Musculoskeletal Research, University of Manchester, 2nd Floor, Stopford Building, Oxford Road, Manchester, M13 9PT UK; Manchester Academic Health Sciences Centre, Institute for Human Development, Royal Manchester Children’s Hospital, 5th Floor Research, Oxford Road, Manchester, M13 9WL UK; Stem Cell and Leukaemia Proteomics Laboratory, School of Cancer and Imaging Sciences, University of Manchester, Manchester, UK

**Keywords:** Juvenile idiopathic arthritis, System biology, Network analysis

## Abstract

Juvenile idiopathic arthritis (JIA) is a clinically diverse and genetically complex autoimmune disease. Currently, there is very limited understanding of the potential underlying mechanisms that result in the range of phenotypes which constitute JIA.

The elucidation of the functional relevance of genetic associations with phenotypic traits is a fundamental problem that hampers the translation of genetic observations to plausible medical interventions. Genome wide association studies, and subsequent fine-mapping studies in JIA patients, have identified many genetic variants associated with disease. Such approaches rely on ‘tag’ single nucleotide polymorphisms (SNPs). The associated SNPs are rarely functional variants, so the extrapolation of genetic association data to the identification of biologically meaningful findings can be a protracted undertaking. Integrative genomics aims to bridge the gap between genotype and phenotype.

Systems biology, principally through network analysis, is emerging as a valuable way to identify biological pathways of relevance to complex genetic diseases. This review aims to highlight recent findings in systems biology related to JIA in an attempt to assist in the understanding of JIA pathogenesis and therapeutic target identification.

**Note:** Throughout this review the original terms are used for the patients JIA or JRA as they occurred within the original publications.

## Background

### Network analysis

**Fig. 1:**
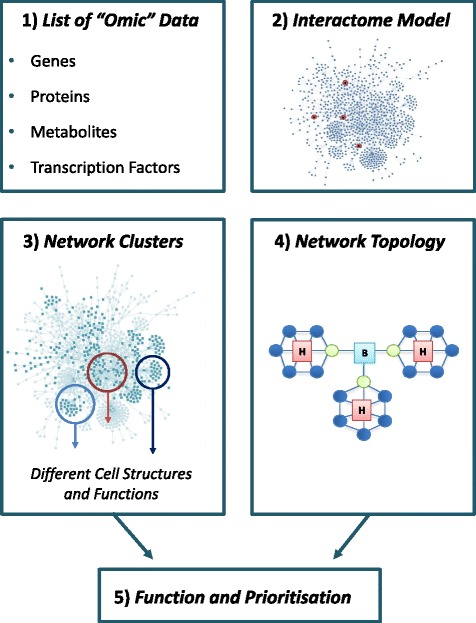
Network biology identifies the relationships between diverse biological components (1). The singular components are then analyzed in a biological system, or interactome model (2) to understand the physical and functional relationships. The subsequent study of sub-networks that might represent biological molecules functionally linked working in a coordinate manner (3) and the topological structure of a network (4) are important to depict and prioritise a specific biological function (5)

The many molecular interactions that occur in living cells and organisms can be represented by networks of genes and proteins. These networks can be linked to disease mechanisms and response to therapy to facilitate understanding and generate new insight. Here we present an introduction as to how networks are generated using ‘omic datasets and show how they can be used to understand both function (molecular phenotype) and prediction/classification using juvenile idiopathic arthritis as an exemplar condition of pediatric rheumatology (Fig. 1).

Network biology has developed as a method to study the many interactions that occur in individual cells; this has facilitated the understanding of the complex biological processes which occur in molecular biology [1]. The recent genome sequencing projects have provided a nearly complete list of human gene products [2] along with the development of connectivity maps between proteins [3] and gene expression (GE) profiles for several tissues [4].

Network biology is able to identify relationships between diverse biological components, including genes, proteins, metabolites and transcription factors [5] (Fig. 1). The singular components are then analyzed in a biological model system to understand physical interactions and functional relationships which can be potentially used to elucidate the pathophysiology of different conditions. The analysis of the structure of biological networks and ‘omic’ methodologies also allows the identification of candidate disease-causing genes.

The aim of this review is to explain how the analysis of biological networks can be carried out, and to illustrate how systems biology is impacting on our understanding of complex genetic diseases, with a particular focus on juvenile idiopathic arthritis (JIA).

### How do we visualize biological network models?

Biological networks are ‘graphs of connected nodes’ which represent biological components connected through edges to represent their specific relationships. These connections vary from physical to functional associations, and from metabolic to regulatory pathways [5] (see Glossary for definitions).

Several models have been used for network analysis [1]. In Bayesian networks, the nodes represent biological variables and the edges conditional probabilities between them. Due to their capacity for showing causal relationship [6], Bayesian networks have been used in several fields of systems biology, including pathway modeling, quality assessment of protein-protein interactions and functional annotation of proteins and GE analysis [1]. However, the commonest representations of biological networks are as “node and edge” diagrams, where “nodes” represent proteins or genes and “edges” represent the known interactions. These can be directed, with an arrow representing “action” or “flow” of information, or un-directed, where connections represent interactions, Fig. 1.

### The analysis of omic datasets using network approaches

Biological networks can be inferred using omic datasets by mapping the data to models of all known protein-protein or genetic interactions (“the human interactome”) and creating a network model related to the condition being examined [7]. An interactome, therefore, is a set of molecular interactions, both with direct and inferred experimental evidence, that are involved with the phenomenon being studied (Fig. 1). Different omic datasets can be combined easily using this approach to allow multi-variate analysis and to increase statistical certainty [8].

To perform a basic analysis of omic datasets, two or more groups are generally compared statistically; however the main problem in this process is that the number of comparisons is large, increasing the likelihood of false positives. This phenomenon, to some extent, can be dealt with by the application of a false discovery rate (FDR) correction to the statistical test (for example Benjamini-Hochberg [9]), however these corrections can be harsh, particularly when small datasets are used, as is often the case in pediatric medicine. The use of network biology to position elements from omic datasets into networks along with all known protein:protein and protein:genetic interactions allows the analysis of these data sets in relation to their position within the human interactome and this feature can additionally increase the confidence in the analysis of omic data [10]. This process is facilitated by the identification of “clusters” within biological networks, which occur when changes in local connectivity arise and consequently network elements coalesce into groups that are associated with biological function [11, 12]. Therefore, network analysis can be used to permit a more error tolerant interpretation of different types of omic datasets as clusters are identified using multiple single elements from the omic dataset [8, 13].

The development of transcriptomics, which includes whole genome transcript data, has also enabled the study of “upstream” regulators in the genome, another approach facilitated by network analysis. Using this method it is possible to group changes in transcriptomic data based on the known action of upstream regulators, such as transcription factors and microRNA (miRNA), allowing the interpretation of integrated data to delineate putative mechanisms [14]. Additionally, expression quantitative trait locus (eQTL) data can be used to integrate genetic and transcriptomic analysis [15].

An example of how transcriptomics has been used in pediatric rheumatology is shown by the work in relation to the identification of interleukin-1 GE changes in children with systemic onset JIA. These observations have resulted in the development of interleukin-1 blockade, which has been utilised to treat systemic onset JIA and related autoinflammatory diseases. Overall, these studies on GE helped in understanding the disease pathogenesis of JIA with important implications for direct therapeutic targeting [16, 17].

### Common software-based methods for network analysis

Several analytic methods for evaluating the relationship between the properties of biological networks can be used to assess biological function [8], as illustrated in Fig. 1, with definitions provided in the Glossary. An example is “cluster modularity”. This refers to the recognition of sub-networks that might represent biological molecules which are functionally linked and working in a coordinate manner to achieve a definite biological outcome [11, 18].

To enable the examination of extensively overlapping network modules the ModuLand algorithm has recently been developed. This applies the concept of community centrality, which is an integrated measure of the influence of the network on one of its nodes or edges [11]. This approach overlaps well with other clustering methods. It has provided an important new analytical tool in that the overlap of network clusters can be quantified, a feature that has a strong correlation to biological function [11].

“Network motif” analysis represents the identification of small networks, related to biological function, that are over-represented when compared with a randomized version of the same network [12]. Other important concepts in network analysis related to function include “node centrality” and “network robustness” [19] along with “network alignment and comparison”, an approach used to describe similarities between independent networks which has been particularly used to study the evolutionarily conserved pathways [20].

It is also possible to compare positions within interactome models to assess whether condition related changes in network connectivity occur at a level greater than that expected by random modeling [21]. This approach has been driven by genome-wide association studies (GWAS) requiring a need to prioritise SNPs based on supportive functional evidence. The Disease Association Protein-Protein Link Evaluator algorithm (DAPPLE) [http://www.broadinstitute.org/mpg/dapple/dapple.php] is a useful tool for such prioritization.

Different software-based methods can be used to generate biological networks. These utilize algorithms to infer the relationship of omic changes with known interactions in the literature or from other databases. Open-access databases include Biogrid [7] and Reactome [22]. The interactome models generated can be then visualized using Cytoscape, which is an open source software platform for the analysis of complex networks [23]. Network analysis is also a process through which to increase confidence in the observations of differential GE by correlation with biological function [24].

Ingenuity Pathway Analysis (IPA) software, widely accessed and reported on by the scientific community, has a network component to its analytical flow based on the functional correlation of highly connected interactome regions with target genes [24]. The IPA network identification algorithm has proved to be very successful but only in the in the context of no more than a few hundred starting genes/proteins. A main feature of the IPA network approach is the ranking of identified networks based on the statistical significance of the associated biological functions. This has limitations as it may not reflect the interactome hierarchy involved in the mechanism.

Analysis of network structure and topological analysis can be derived when using the Cytoscape software platform [23]. Using this approach it is possible to generate a minimal essential network [MEN] [25, 26]. A MEN represents the most functionally relevant elements of an interactome model and can be used to assess biological function [27].

## Disease networks

Networks of biological interactions involved in disease have been constructed and their properties have been compared to gain insight into the pathogenesis of human disease [28, 29]. A knowledge of the network properties of “disease genes” can inform key aspects when researching complex diseases: it allows for the identification of new disease genes; identifies new drug targets; identifies biomarkers and enhances understanding of the biological significance of disease-associated DNA variations from GWAS or next-generation sequencing studies [28].

Recently, the “human diseasome” has been generated by using network biology and combined datasets of all known disease-gene associations [30, 31]. This has been created on the principle that there is a consistent relationship between disease-causing genes and their products, and developed on a conceptual framework which systematically links all recorded genetic disorders (the “human disease phenome”) with the current complete list of known disease-causing genes (the “human disease genome”) [31]. From “the human diseasome” it has become apparent that genes contributing to a common disorder exhibit: (i) an increased propensity for their products to co-interact through protein-protein interactions (ii) have a tendency to be co-expressed in specific tissues and (iii) tend to share common cellular and functional characteristics, as annotated in the Gene Ontology [1, 32].

## Network analysis and JIA

To date, only a limited number of studies have employed a network analysis component to research involving JIA patients. The contributions each of these has made to further our understanding of JIA pathogenesis is discussed:

### Role of neutrophils in the pathogenesis of polyarticular JRA

GE arrays and RNA seq have been used to examine the function of neutrophils in JIA [33, 34]. Jarvis et al. [33] used computer modeling from 25 newly diagnosed rheumatoid factor negative (RF-ve) polyarticular JRA patients. Fourteen of the children were studied on more than one occasion to identify changes in GE patterns in response to therapy. Ten healthy controls (ages 18–30 years) were also included. A computer model of differentially expressed genes in the RF-ve polyarticular JRA cases and control neutrophils was developed using the PathwayAssist software. This identified up regulation of the S100 proteins, a family of low molecular weight proteins implicated in a variety of growth and immune functions, in the patient group and revealed clusters of genes independently or interdependently regulated by interleukin-8 or interferon-γ. This network based analysis also showed significant associations between differentially expressed genes and the regulation of fundamental metabolic processes such as H_2_O_2_ production and calcium influx.

### Implications for JIA therapy

In an innovative approach Frank et al. [35] utilized network analysis to examine the feasibility of using GE profiling as a first step in understanding the structure of pathogenic networks related to childhood onset rheumatic diseases. RF-ve polyarticular JIA patients (*n* = 14), juvenile dermatomyositis patients (*n* = 17) and 11 healthy children were included in the study. GE differences in neutrophils and in peripheral blood mononuclear cells (PBMCs) were examined. To better understand the potential functional interactions between the products of genes that were differentially expressed in the childhood-onset rheumatic diseases relative to the healthy controls these genes were analyzed using IPA software. Of the 128 genes that were differentially expressed in PBMCs from JIA patients relative to controls seven networks were identified that each contained 12 or more differentially expressed genes. High connectivity was found for tumor necrosis factor alpha (TNFα) and interferon gamma (IFN-γ). Similarly, of 60 genes differentially expressed in neutrophils between JIA patients and the controls IPA identified four networks that each contained at least nine differentially expressed genes. NF kappa B and the kinases ERK, p38MAPK and MAPK14 showed the highest connectivity.

Thus, the network analysis showed evidence of hub and node structures, indicative of scale-free networks, previously described for normal metabolic processes, and identified the long-suspected pathologic hubs centering around TNFα and IFN-γ for JIA. One of the most important features of scale-free networks is their relative resistance to perturbation when peripheral nodes are targeted. Only alterations in the hubs results in significant alteration in the network [25, 36].

The most promising therapeutic targets are those directed at pathologic hubs. Even if a gene shows strong differential expression between children with disease and control children, that gene is unlikely to be a promising therapeutic target if it is a peripheral node. Proof-of-concept for this in JIA comes from TNF inhibitors that have, over the past 20 years, been highly successful in the therapeutic management of JIA patients. TNFα is a prominent hub in the pathology-associated metabolic network in both neutrophils and PBMCs determined by Frank et al. [35].

### Identification of biomarkers of therapeutic response

Network analysis, using IPA, was used by Knowlton et al. [37] to aid the identification of biomarkers for predicting response to therapy in RF-ve polyarticular JIA patients. When children with active disease were compared with children who had achieved clinical remission while receiving medication 23 differentially expressed genes were found, 22 of which were over-expressed in the children with active disease. Network analysis, derived via IPA, revealed a single network, central to which is insulin. The functional significance of insulin to JIA aetiopathogenesis, however, has not been determined.

Most recently Du et al. have shown that methotrexate therapy in JIA is associated with mathematically defined re-ordering of gene expression networks in children who respond inadequately to therapy [38].

### JIA subtypes and other autoimmune conditions

IPA was also used by Barnes et al. to identify differences in peripheral blood GE in different subtypes of JIA patients [39]. Forty-six pathways that were overrepresented in the PBMCs of JIA patients were compared with that found in healthy children. The GE differences and relative contributions of each pathway differed between the JIA subgroups. The number of over-represented pathways was greatest for the systemic onset JIA patient subgroup (*n* = 34). This included up-regulation of innate immune pathways, the peroxisome proliferator-activator receptor (PPAR) signaling pathway and the complement system and coagulation cascade.

More recently, in an attempt to identify common and specific signatures of GE and protein-protein interactions in autoimmune diseases [40], GE and protein-protein interaction data from six autoimmune diseases were compared, including 26 children with JRA. These included 15 individuals with polyarticular course disease, 3 with pauciarticular onset, 9 with polyarticular onset and 3 with systemic onset disease. Information regarding rheumatoid factor (RF) status was missing on 7 children, all of which were classified as having RF-ve disease. From this heterogeneous cohort of arthritis subgroups a cluster of 43 proteins specific to JRA was identified. Multiple pathways were shared between JIA patients and those from the other autoimmune conditions. These included NF kappa B, IL-2, IL-6 and B cell receptor signaling pathways. Hierarchical clustering however revealed the GE signature of JRA patients to be relatively different from the signatures of the other diseases. The authors conducted their analysis using a multi-step approach. A large-scale data set of human protein-protein interactions (PPI) network was used to compute, for each gene in each of the 6 autoimmune diseases investigated, a p-value based on the proteins that have PPIs with the protein it encodes and their expression levels. The focus was to attempt to detect post-transcriptional regulatory changes in diseases based on mRNA measurements. For this they invented the PPI *p*-value approach [40]. Replication of the findings, using the application of the PPI *p*-value, needs to be undertaken.

## JIA – age specific features

The individual subgroups recognized under the JIA umbrella, defined by The International League of Associations for Rheumatology (ILAR) classification, are phenotypically varied.

The typical age of disease onset also depends on subtype (see JIA subtypes, Table 1). This age-specific variation in disease onset may be critical to understanding disease aetiology and have implications for appropriate treatment strategies.

**Table 1:** Juvenile idiopathic arthritis subtypes show specific age ranges for disease onset

Categories	Characteristics	% of total	Onset age	Sex ratio (F:M)
Systemic onset	Arthritis and daily fever ≥ 3 days, accompanied by at least one of the following: evanescent (non-fixed) erythematosus rash, generalised lymph node enlargement, hepatomegaly or splenomegaly (or both), serositis	4–17	Throughout childhood	1:1
Oligoarticular	Arthritis affecting 1–4 joints during the first 6 months of disease	27–60	Early childhood (peak 2–4 years)	5:1
Persistent	Arthritis affecting < 4 joints throughout the disease course	40		
Extended	Arthritis affecting > 4 joints after the first 6 months of disease	20		
Polyarticular	Arthritis affecting > 5 joints during the first 6 months of disease			
Rheumatoid factor positive	Two or more positive tests for rheumatoid factor at least 3 months apart	2–7	Late childhood or adolescence (peak 12–14 years)	3:1
Rheumatoid factor negative	Tests for rheumatoid factor negative	11–30	Early peak 2–4 years and late peak 6–12 years	3:1
Juvenile psoriatic arthritis	Arthritis and psoriasis, or arthritis and at least 2 of the following: dactylitis, nail pitting or onycholysis, psoriasis in first degree relative	2–11	Late childhood or adolescence	1:0.95
Enthesitis related arthritis	Arthritis and enthesitis, or arthritis or enthesitis with at least 2 of the following: sacroiliac joint tenderness or inflammatory lumosacral pain (or both), HLA-B27 antigen positive, onset in boy over 6 years old, acute anterior uveitis, HLA-B27 associated disease in first degree relative	1–11	Early peak 2–4 years and late peak 6–12 years	1:7
Undifferentiated arthritis	Arthritis that fulfils criteria in no specific category or meets criteria for more than one category	11–21		

Adapted from Prince et al. [43]

Stevens et al. [36] employed network analysis of GE data and have determined evolutionarily conserved tissue-independent pathways associated with GE and child development in multiple tissues. Specifically, using cells of lymphoid origin from normal children, the expression of 688 genes (ANOVA FDR modified *p*-value, q < 0.1) was associated with age, and subsets of these genes formed clusters that correlated with the phases of growth: including infancy, childhood, puberty and final height. Network analysis on these clusters identified evolutionarily conserved growth pathways (NOTCH, VEGF, TGFβ, WNT and the glucocorticoid receptor) and the same observations were confirmed in other tissues studied, suggesting the existence of a tissue-independent genetic program for human growth and development. Overall, these findings highlight the existence of age-dependent GE profiles. These are most likely to be relevant to the appropriate selection of genes and pathways as potential biomarkers of disease, or as age appropriate drug targets, in age-related phenotypes, such as JIA.

Barnes et al. also described biological similarities based on age definition in oligoarticular and polyarticular subtypes of JIA [41]. Earlier observations made by Hollenbach et al. [42], which showed that HLA disease associations with DRB1;DQA1;DQB1 haplotypes conferred variable risk according to JIA subgroup and age at disease onset, had been made based on stratification of the JIA patient population by age of disease onset at or after 6 years of age. Utilizing this same age classification, Barnes et al. investigated GE profiles from PBMCs of JIA patients and healthy controls [41]. Principal component based analysis confirmed age at disease onset to be an important characteristic for oligoarticular and RF–ve polyarticular JIA subgroups. The differential GE patterns indicated that pathologic mechanisms differ between patients with early onset (<6 years) disease compared to those with late-onset (≥6 years) disease. This age correlated variability in GE could have important implications for treatment interventions in JIA patients.

### Network analysis of age related GE in JIA

**Fig. 2:**
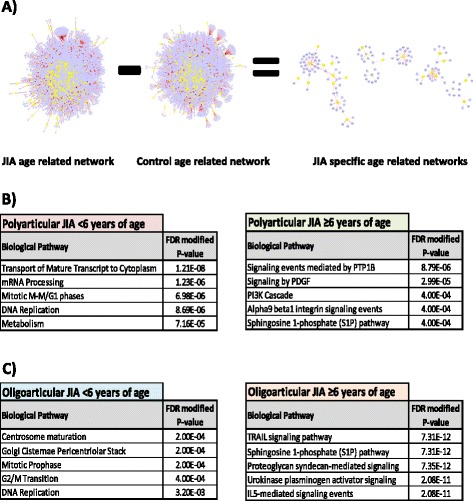
Network analysis of age-related gene expression in JIA. Age-related gene expression in JIA and control pediatric groups was derived from published sources (JIA & Controls: Barnes et al. [41], GSE 20307; Controls: Stevens et al. [36]). Age groups: Less than 6 years of age [<6], polyarticular JIA *n* = 16 [657 genes], oligoarticular JIA *n* = 24 [530 genes], controls *n* = 63 [438 genes]; greater than or equal to 6 years of age [≥6], polyarticular JIA *n* = 28 [512 genes], oligoarticular JIA *n* = 16 [811 genes], controls *n* = 71 [415 genes]. (**a**) Interactome network models inferred from age-related gene expression were generated using the BioGRID database (http://thebiogrid.org/; version 3.2.103); yellow = protein derived from gene with age-related change in expression, blue = protein inferred to interact in association with age-related gene expression. Interactome models were generated for the <6 and ≥6 age groups for polyarticular and oligoarticular JIA [41] along with the control group (combined data from Barnes et al. [41] & Stevens et al. [36]). To generate JIA specific age-related interactome models the control networks were “subtracted” from the JIA derived networks using the “network differences” plugin within Cytoscape 2.8.3 [23]. JIA specific age-related gene expression identified was used to determine associated biological pathways (hypergeometric test with Benjamini-Hochberg false discovery rate modification [FDR]; performed using WEB-based GEne SeT AnaLysis Toolkit [Webgestalt; http://bioinfo.vanderbilt.edu/webgestalt/]). Top biological pathways associated with age-related gene expression ranked by FDR modified p-value (**b**) specific for polyarticular JIA and (**c**) specific for oligoarticular JIA

We have extended the observations of Hollenbach et al. and of Barnes et al. (Fig. 2). Specifically, interactome models were generated for the early onset (<6 years) and the late onset (≥6 years) disease age groups for RF-ve polyarticular and oligoarticular JIA [41] patients, along with a control group using combined data from Barnes et al. 2010 [41] and Stevens et al. 2013 [36]. This JIA specific age-related GE was used to determine associated biological pathways which were either specific for RF-ve polyarticular JIA or for oligoarticular JIA (Fig. 2). For the age group < 6 years, for both these JIA subtypes, biological pathways related to DNA replication and cell cycle were the most statistically significant pathways identified (Fig. 2), whereas, for the older age group (≥6 years) intracellular signalling pathways, including growth factors and interleukins, were predominant. These findings reveal age-related GE profiles in JIA, strengthening the notion that different pathological processes underlie the age of disease onset and the JIA subgroup that manifests. The relevance of this age-related GE in the successful therapeutic management of JIA needs to be explored.

## Conclusion

To date there has been limited application of systems biology based approaches to JIA. Where network analysis has been applied to JIA it has supported the identification of specific biological pathways associated with pathology, the identification of markers of response to therapy and also helped with the clarification of the relationship of JIA to other autoimmune diseases. Furthermore, network approaches to transcriptomic datasets from JIA patients has shown a correlation between age and variability in GE that may be critical to our understanding of individual JIA subgroups.

Applying network analysis to JIA, to integrate emerging forms of data from multiple platforms, has the potential to identify key pathways of importance. This in turn can expedite our understanding of disease mechanisms and reveal interactions that should be prioritised for therapeutic benefit.

## Abbreviations

ACR, American College of Rheumatology; DAPPLE, The Disease Association Protein-Protein Link Evaluator algorithm; eQTL, expression quantitative trait locus; FDR, false discovery rate; GE, gene expression; GWAS, genome-wide association studies; IFN-γ, interferon gamma; ILAR, The International League of Associations for Rheumatology; IPA, Ingenuity Pathway Analysis; JIA, juvenile idiopathic arthritis; JRA, juvenile rheumatoid arthritis; MEN, minimal essential network; miRNA, MicroRNA; PBMCs, peripheral blood mononuclear cells; PPAR, peroxisome proliferator-activator receptor; PPI, protein-protein interactions; RF, rheumatoid factor; RF-ve, rheumatoid factor negative; SNPs, single nucleotide polymorphisms; TNFα, tumor necrosis factor alpha.

## Glossary

Systems Biology: Integration of complex data in biological systems from diverse experimental sources using interdisciplinary tools.
Network Biology: Biology related to interactions between multiple genes and/or proteins.
Network Analysis: Studies the relationship between the structural properties of a network and biological function.
Interactome: Biological network representing a whole set of direct or indirect interactions related to a specific biological function.
Cluster Modularity: Distinct grouping of protein-protein or protein-gene interactions within a network.
Node: A protein or gene positioned within a network.
Hub: A highly connected node within a network.
Node Centrality: Measures the centrality of nodes, with the identification of which nodes are more “central” than others. Degree centrality of a node refers to the number of edges attached to the node.
Network Robustness: It is a mathematical description of how the integrity of a network responds to the random removal of single nodes.
Network Motifs: Recurrent and statistically significant sub-graphs or patterns within a network.
Network Alignment and Comparison: Used to describe similarities between independent networks.
